# Supplementary material for: Disruption of Cav1.2-mediated signaling is a pathway for ketamine-induced pathology
Source: Nat Commun. 2020 Aug 28;11:4328. doi: 10.1038/s41467-020-18167-4 (PMC7455701; doi:10.1038/s41467-020-18167-4)
Supplement: Supplementary file 1 — Supplementary Information [file 41467_2020_18167_MOESM1_ESM.pdf]

Supplementary Information

Disruption of Cav1.2-mediated signaling is a pathway for ketamine-induced pathology

Chen et al.

Supplementary Table 1. List of primers for RT-PCR

Gene	5' primer sequence	3' primer sequence
Creb1	CAGATGTGGTTTTGAAGCTCG	TGGGATTAAAGGTACGTGCTAC
Creb2	GAAGAGGTCCGTAAGGCAAG	CAAACACAGCAACACAAGACT
Creb3	CAGGGCAGGTATTCAGGTTAG	CATCCTAACAGAGCACGTGAG
Creb5	CCAGAACAGTCAGACTCCTTG	CTGTGGTTTGGGTTTGATCTG
MEF2a	TGCAAACTCCTTGTAGGTCTG	ATGGGTACACGTTTCTCTGC
MEF2b	GTTCCCGAAGTCTCAGGTTC	GTCCTTGGGCTTTATTGATGC
MEF2c	CGGTAATTGTAGGAACACGCC	CATACGCCAATGATATGCCTG
MEF2d	CGGCTGGATACTTGGACATT	AACAGGACATGGAGCAGAAG
FoxO1	GGTGTCAGGCTAAGAGTTAGTG	TGAAGGGCATCTTTGGACTG
FoxO3	CGTTGTTGGTTTGAATGTGGG	GGTTTTCTCTGTAGGTCTTCCG
FoxO4	TGGAGTGCGACATGGATAAC	AAAAGTGGATAAGGACAGGCC
FoxO6	ATGACACGTTTCCCTCACTG	GACTGGTTAAGATGGGAGACTG
Nfatc1	CTCAAAGAAAAGCGAAGCCTG	CAAAAGGCGAGCAAGTGATG
Nfatc2	ACCCAGTCAAAACCTACACG	TCAGAGCAAAGGGAGATTTCG
Nfatc3	GCACTGGCTGAAGACTTAATG	GAATCTGTTGCTCCTACCCTG
Nfatc4	GAGTGAGATCATTGGCCGAG	CCAGCACCCCAGTTAAGAG
Nfatc5	GCAGTCAGAACATGGAAAAGATTG	CCACAGTTCTCAGGATATCTTGG
c-fos	TCCAGTCCTCACCTCTTCC	AAGAGAAAAGAGACACAGACCAG
c-jun	GTTGAAAGCTGTATGAAGTGGC	TCAAAGTTGGAAGGAGACACC
Cav1.2	CTTGAAATCCACCTACCAGACC	CTTGAAATCCACCTACCAGACC
NR1	CAACCTGACTTCCACCTCTATC	ACTAGGGCAGTGAAGGCTAG
SDHA	CTTGAATGAGGCTGACTGTG	ATCACATAAGCTGGTCCTGT

Supplementary Figures

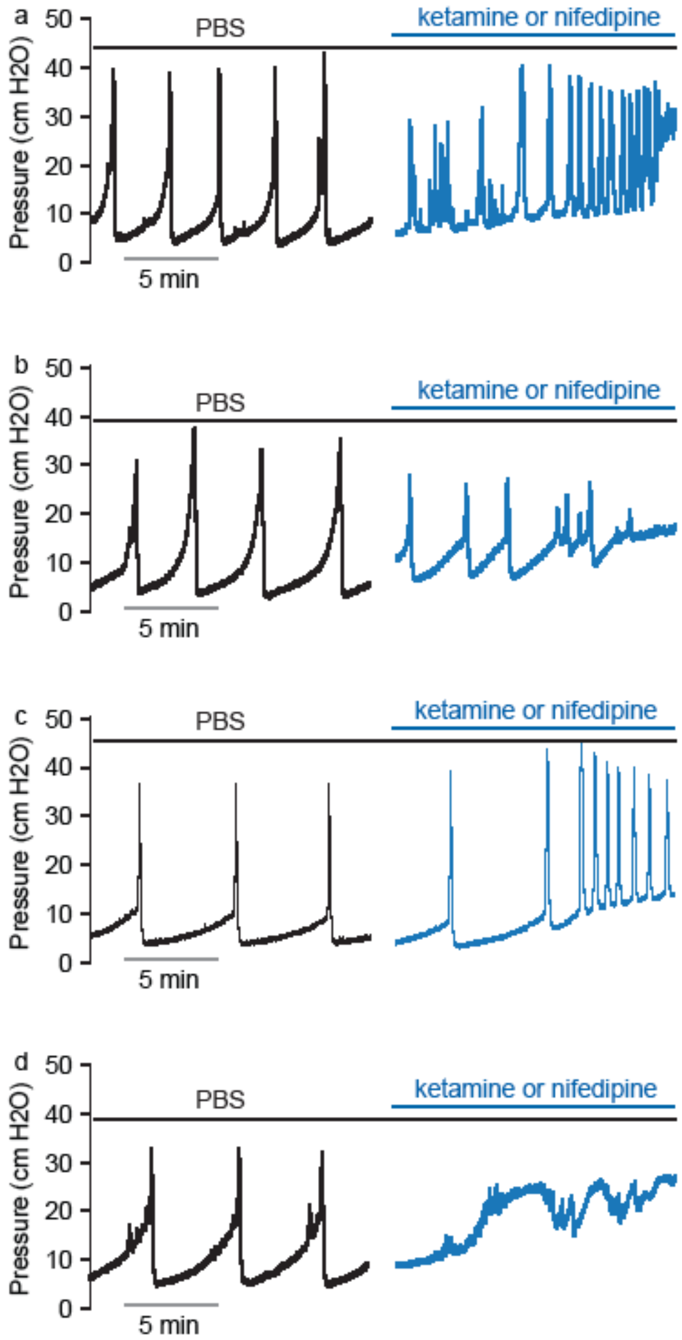

Supplementary figure 1. Ketamine and nifedipine induce a ketamine cystitis-like cystometrogram (CMG) phenotype. Four representative traces of urodynamic changes before and after infusion of ketamine or nifedipine. The urodynamic changes of elevated basal pressure and increased frequency of contractions indicate bladder

spasm. These bladders usually lose ability to void soon after the recording period shown, but even then can be rescued by exposure to Bay k8644 (200 nM) (see Figure 10). These changes in mouse bladder might resemble the elevated voiding frequency that characterize painful human ketamine cystitis.

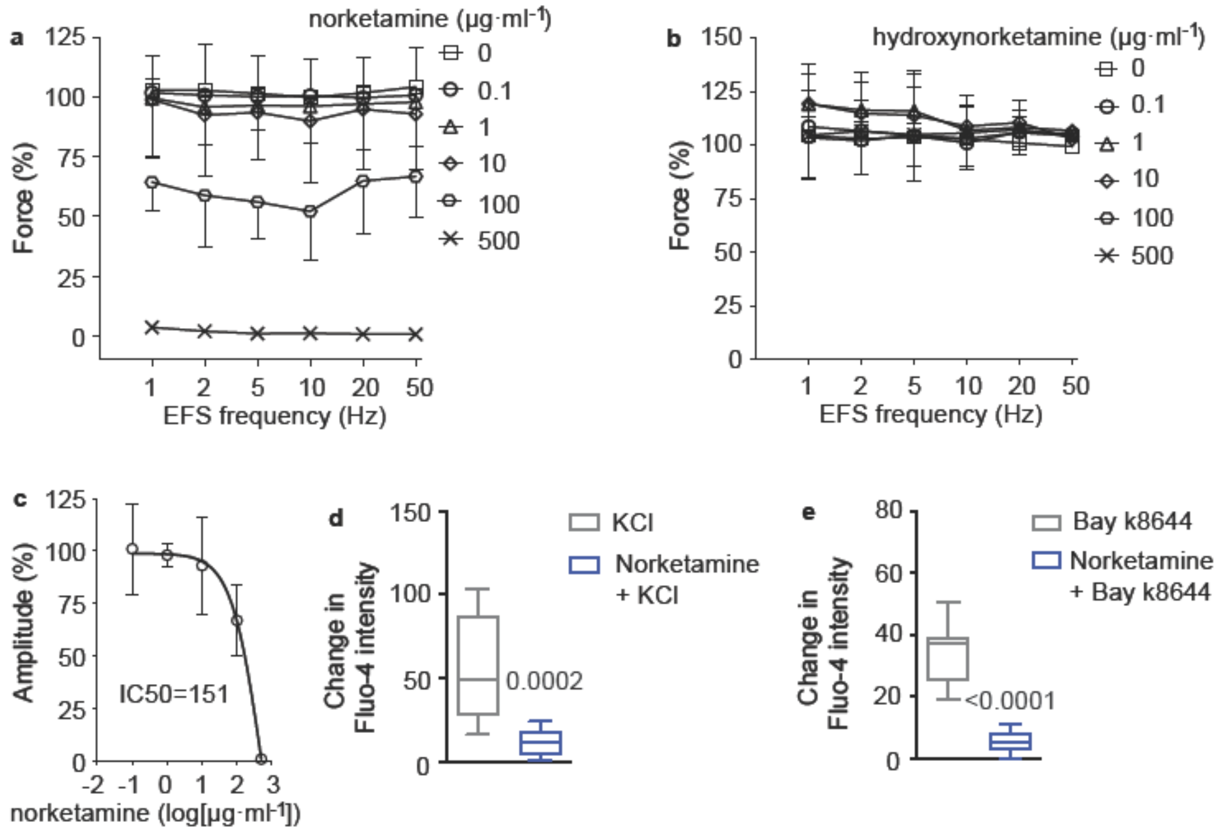

Supplementary figure 2. Ketamine metabolite norketamine dose-dependently inhibits BSM contraction and calcium influx. (a,c) Norketamine dose-dependently inhibited EFS stimulated BSM contraction force, with IC_{50} of ~150 μg ml^{-1} (n=8 BSM strips). However, hydroxynorketamine did not inhibit BSM contractility (b, n=8 BSM strips). Data are presented as mean values ± SD. (d,e) KCl-stimulated (100 mM, n=14 BSM cells) and Bay k8644-stimulated (10 nM, n=20 BSM cells) mouse BSM

calcium influx was inhibited by norketamine (100 μg ml^{-1}) pretreatment (n=18, 14 BSM cells respectively). Data are shown as box and whiskers, center line median value, box represents 75% of the data, and whiskers indicate minimum and maximum values. Data are analysed by two tailed Student's t test. $P < 0.05$ is considered to be significant and P values are given above the bars. Source data are provided as a Source Data file.

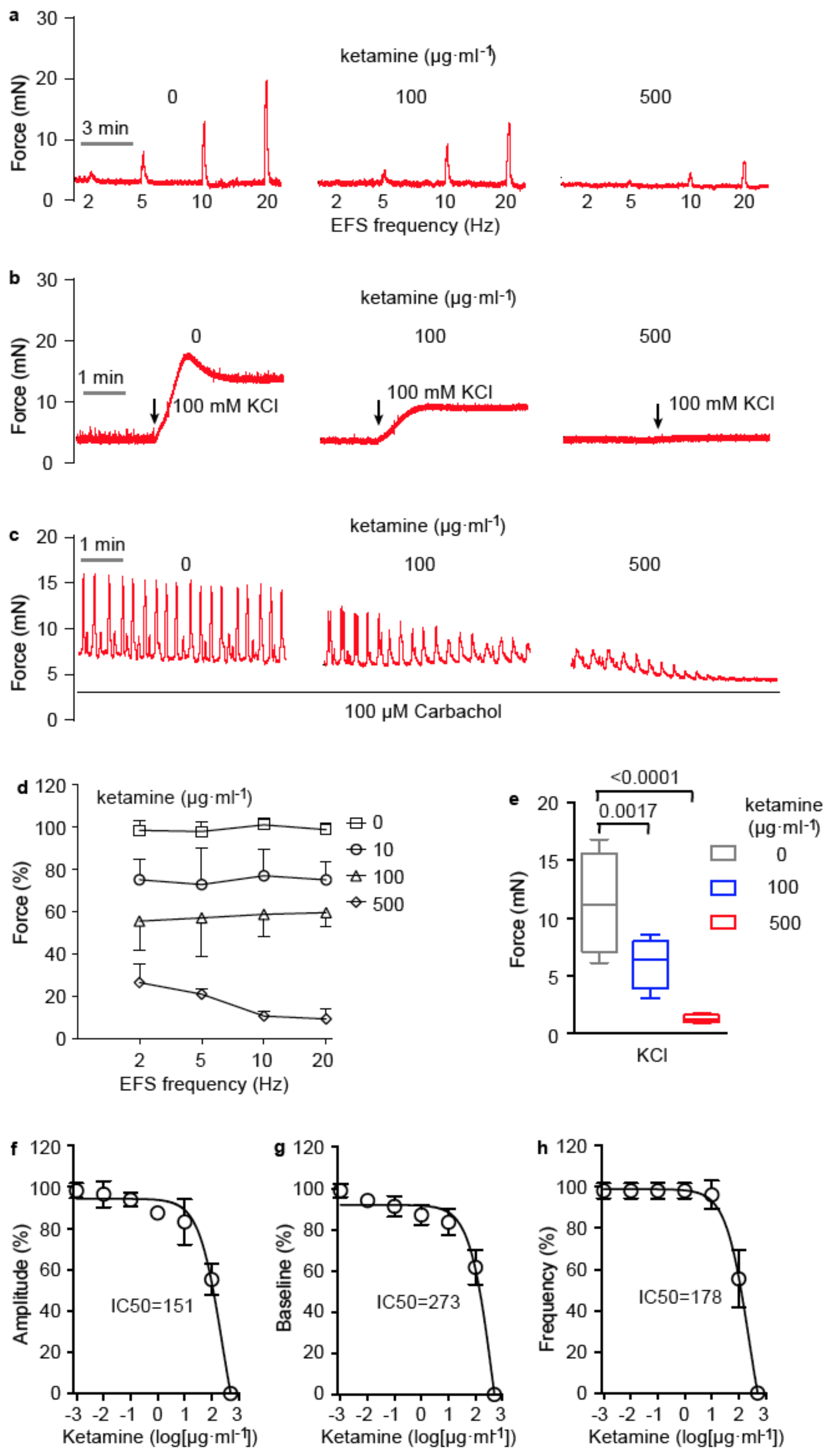

Supplementary figure 3. Ketamine inhibits gastric smooth muscle contraction.

As shown in representative traces of mouse stomach smooth muscle contraction in

response to EFS (a; summarized for n=7 muscle strips in panel d, with data presented as mean values ± SD.), to KCl (b, summarized for n=8 muscle strips in panel e, with data shown as box and whiskers, where center line is median value, box represents 75% of the data, and whiskers indicate minimum and maximum values, and analysis was by one-way ANOVA with Bonferroni's post-hoc tests) and to carbachol (c, summarized for n=8 muscle strips in panels f-h, with data presented as mean values ± SD.). $P < 0.05$ is considered to be significant and P values are given above the bars. Source data are provided as a Source Data file.

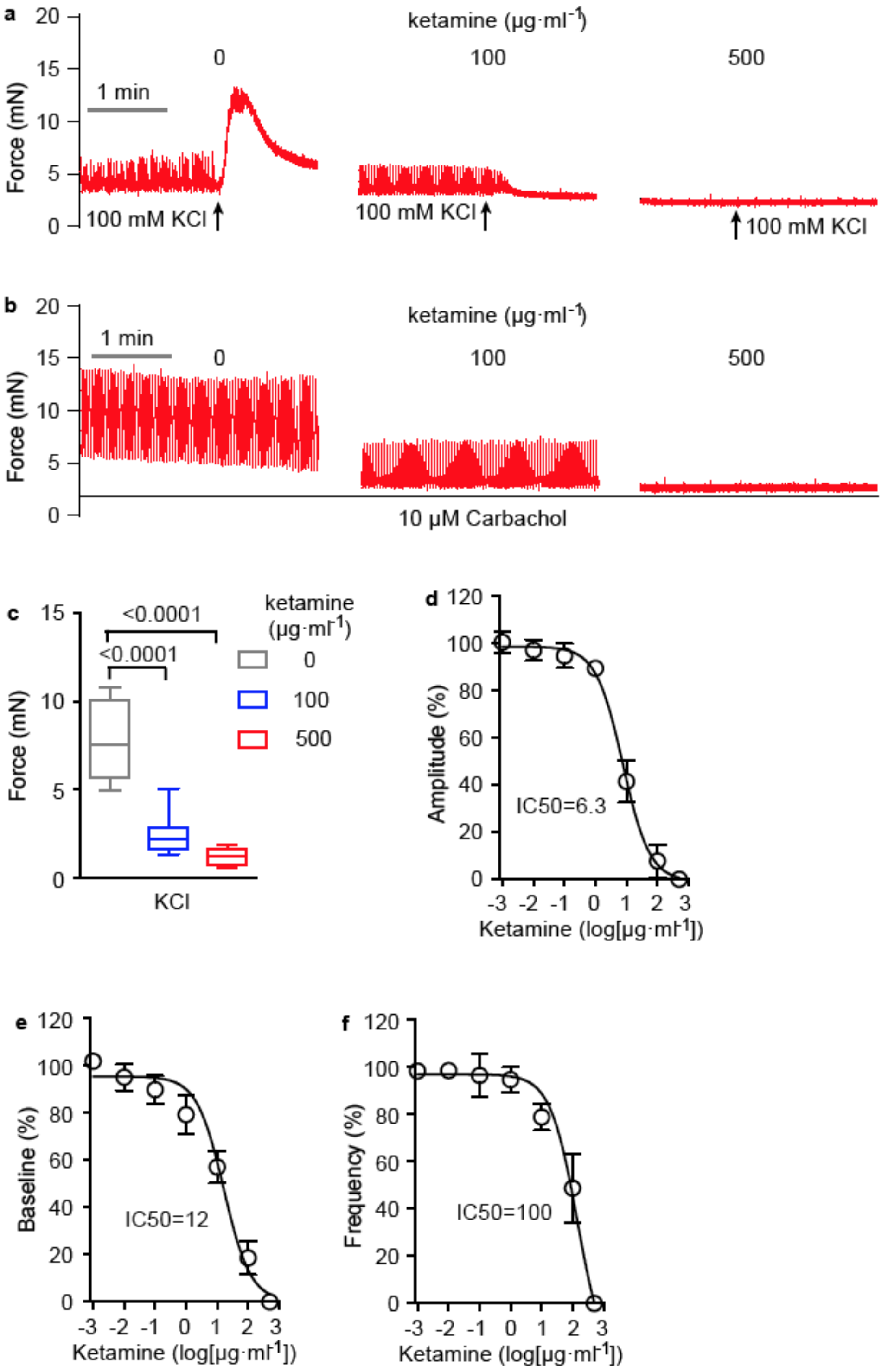

Supplementary figure 4. Ketamine inhibits jejunal smooth muscle contraction. a, representative traces of KCl-induced mouse jejunal smooth muscle contraction, summarized in c (n=10 muscle strips, where data are shown as box and whiskers,

center line is median value, box represents 75% of the data, and whiskers indicate minimum and maximum values. Analysis was by one-way ANOVA with Bonferroni's post-hoc tests, P < 0.05 is considered to be significant and P values are given above the bars). b, representative traces of rhythmic spontaneous contraction, summarized in d, e, and f (n=8 muscle strips). Both types of contraction were dose-dependently inhibited by ketamine. Data are presented as mean values ± SD. Source data are provided as a Source Data file.

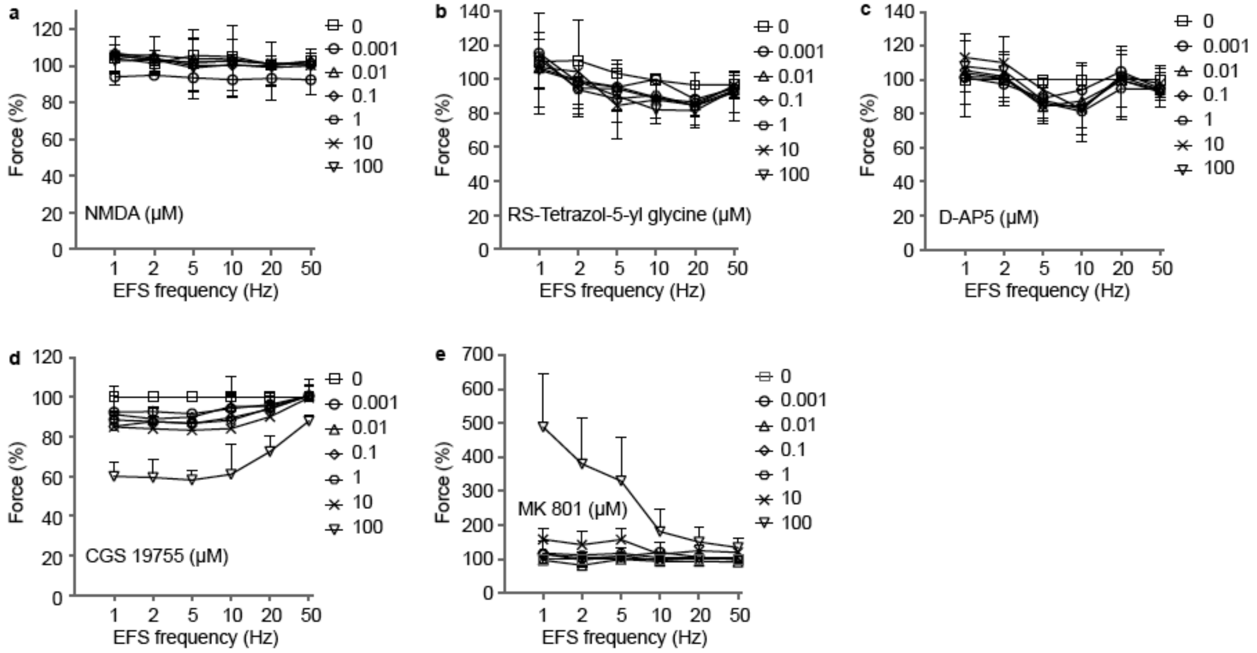

Supplementary figure 5. Neither agonists nor antagonists of NMDAR regulate BSM contractile force. BSM strips were subjected to EFS following pretreatment for 15 min either with NMDAR agonists NMDA (a, n=8 BSM strips) or RS-Tetrazol-5-glycine (b, n=8 BSM strips), or with antagonists D-AP5 (c, n=8 BSM strips), CGS19755 (d, n=7 BSM strips), or MK801 (e, n=8 BSM strips). Contraction forces

were normalized to the responses before pretreatment with agonists or antagonists. The highest tested concentration of competitive NMDAR antagonist CGS 19755 did inhibit contraction at low stimulation frequency. In contrast, non-competitive NMDAR antagonist MK 801 at the highest tested concentration enhanced contractile force only at EFS frequency <u><</u>5Hz. Data are presented as mean values ± SD. Source data are provided as a Source Data file.

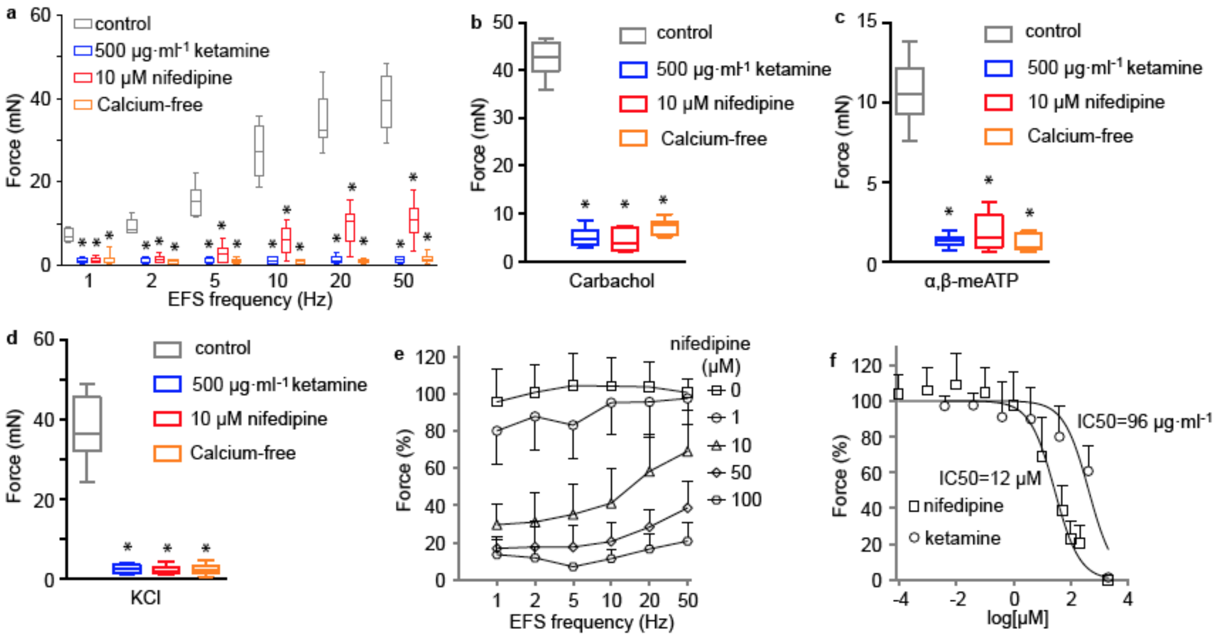

Supplementary figure 6. Ketamine and nifedipine inhibit Ca^{2+}-dependent BSM contraction. Both ketamine and nifedipine inhibit BSM contraction induced by EFS (a, n=10 BSM strips), by 10 μM carbachol (b, n=9 BSM strips), by 10 μM α,β-meATP (c, n=10 BSM strips), and by 50 mM KCl (d, n=10 BSM strips). Data are shown as box and whiskers, center line is the median value, box represents 75% of the data, and whiskers indicate minimum and maximum values. Analyses are by two tailed

Student's t test. * indicates P < 0.0001. e, nifedipine concentration-response curves for inhibition of EFS-stimulated BSM contraction (n=15 BSM strips). Data are presented as mean values ± SD. f, Inhibition of BSM contraction by ketamine (n=12 BSM strips) and nifedipine (n=15 BSM strips). Data analyzed by non-linear curve fit are presented as mean values ± SD. Source data are provided as a Source Data file.

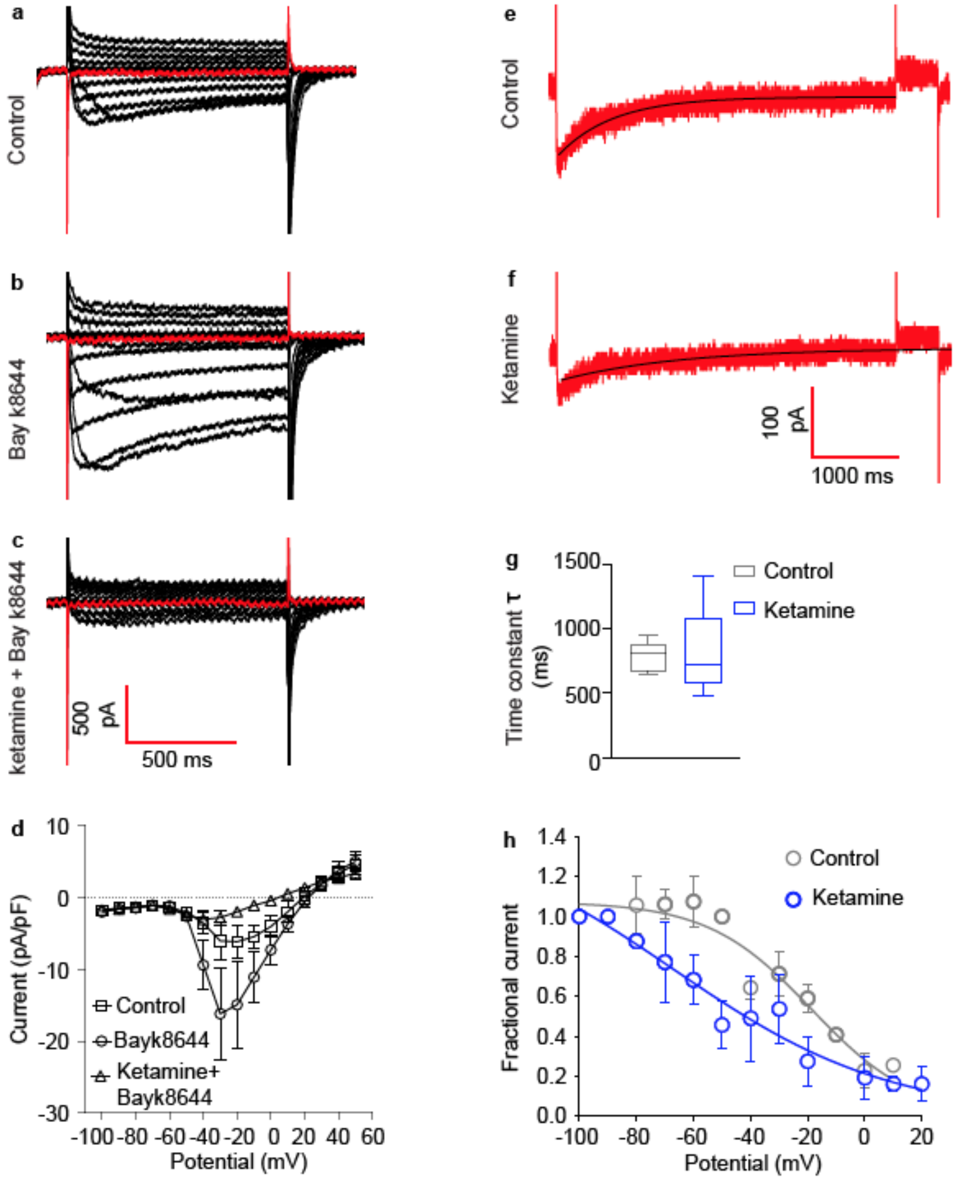

Supplementary figure 7. Ketamine inhibits Bay K8644-induced current. Representative whole cell currents recorded from freshly isolated BSM cells treated with vehicle (a, control, n=5 BSM cells), 100 nM Bay k8644 (b, n=5 BSM cells), and 500 μg/ml ketamine + 100 nM Bay k8644 (c, n=5 BSM cells). d shows summarized data indicating complete inhibition of Cav1.2-mediated current by ketamine (n=5 BSM cells). Data are presented as mean values ± SD. Representative traces of Cav1.2 inactivation in the absence (e, n=5 BSM cells) and presence of 100 μg ml^{-1} ketamine (f, n=5 BSM cells). g, summarized data showing no significant difference between time constants (τ) of single exponential-fit inactivation curves, in absence and presence of ketamine. Double exponential fits failed to increase goodness of fit, and revealed no significant ketamine-dependent differences in rapid or slow inactivation τ values (rapid inactivation of Ba^{2+} currents is greatly attenuated compared to that of Ca^{2+} currents). Data are shown as box and whiskers, center line is the median value, box represents 75% of the data, and whiskers indicate minimum and maximum values. h. is the summarized data of relative peak current (I/I_{max}) fit to a Boltzmann equation, showing ketamine inhibition of BSM cell Cav1.2 activity is mediated in part by inducing a left-shift in voltage-dependent inactivation. Data are presented as mean values ± SD. Source data are provided as a Source Data file.

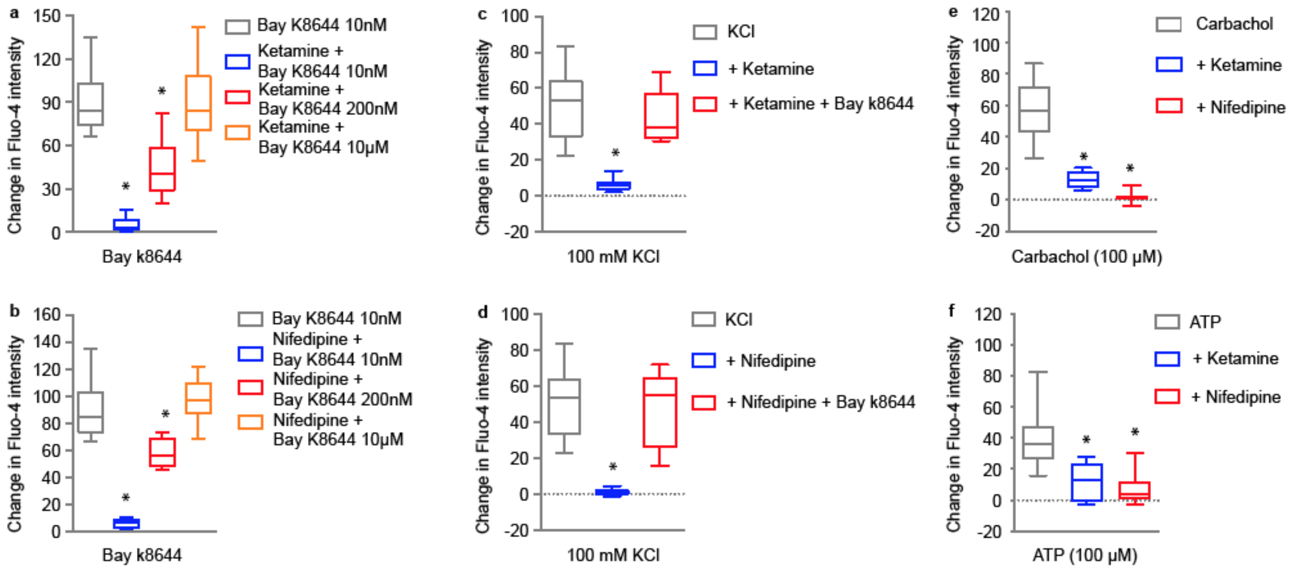

Supplemental figure 8. Ketamine and nifedipine inhibit Cav1.2-mediated calcium influx in human BSM cells. a, Ca^{2+} influx (assessed by Fluo-4 fluorescence intensity) induced by 10 nM Bay k8644 (n=19 BSM cells) was inhibited by 100μg ml^{-1} ketamine (n=24 BSM cells), and that inhibition was partially or completely reversed by increasing Bay k8644 concentration to 200 nM (n=23 BSM cells) or to 10 μM (n=21 BSM cells). b, Ca^{2+} influx induced by 10 nM Bay k8644 (n=19 BSM cells) was inhibited by 10 μM nifedipine (n=22 BSM cells), and that inhibition was partially or completely reversed by increasing Bay k8644 concentration to 200 nM (n=16 BSM cells) or to 10 μM (n=20 BSM cells). c, 100 mM KCl-induced Ca^{2+} influx in human BSM cells (n=15 BSM cells) was inhibited by 100 μg/ml ketamine (n=39 BSM cells) and reversed by addition of 200 nM Bay k8644 (n=14 BSM cells). d, 100 mM KCl-induced Ca^{2+} influx in human BSM cells (n=15 BSM cells) was inhibited by 10 μM nifedipine (n=39 BSM cells), and that inhibition was completely reversed by 200 nM Bay k8644 (n=14 BSM cells). e, 100 μM carbachol-induced Ca^{2+} influx in human BSM cells (n=33 BSM cells) was inhibited by 100 μg ml^{-1} ketamine (n=19 BSM cells) and by

10 μM nifedipine (n=30 BSM cells). f, 100 μM ATP-induced Ca^{2+} influx into human BSM cells (n=29 BSM cells) was inhibited by 100 μg ml^{-1} ketamine (n=20 BSM cells) and by 10 μM nifedipine (n=21 BSM cells). Data are shown as box and whiskers, center line is the median value, box represents 75% of the data, and whiskers indicate minimum and maximum values. Data are analysed by two tailed Student's t test. * indicates P < 0.0001. Source data are provided as a Source Data file.

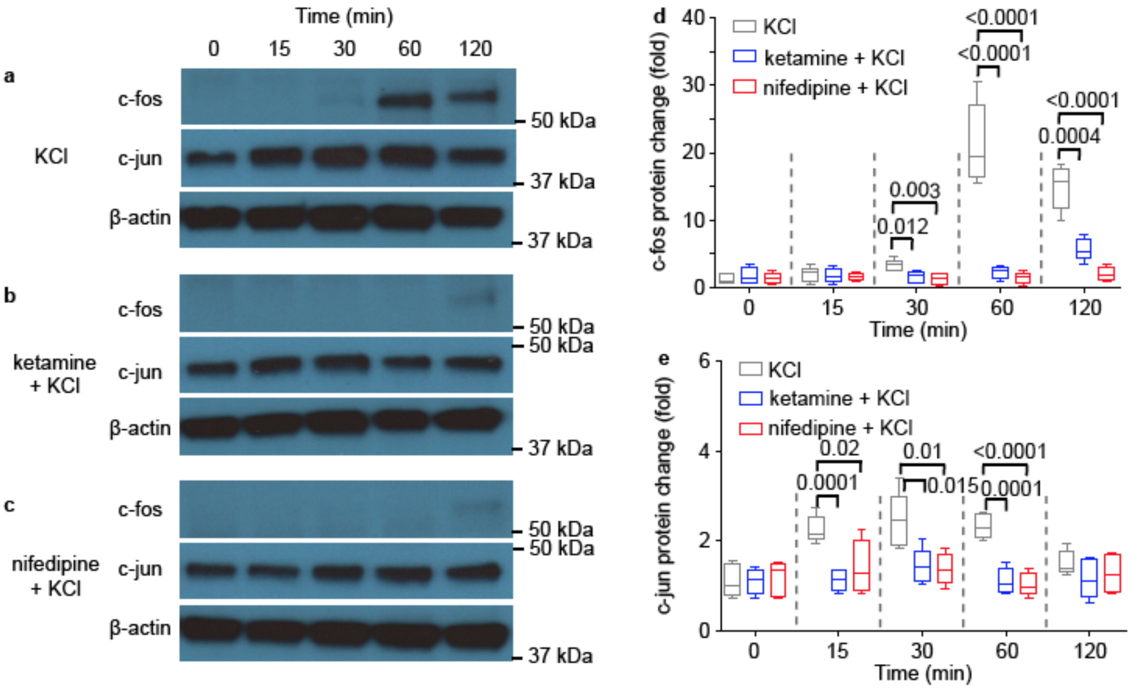

Supplementary figure 9. Ketamine reduces Cav1.2-stimulated increases in levels of c-fos and c-jun in human BSM cells. Primary cultured human BSM cells were subjected to Cav1.2 activation by 50 mM KCl, then lysed at the indicated times for immunoblot analysis of c-fos and c-jun. a, b, and c immunoblots show c-fos and c-jun from BSM cells treated with KCl, 100 μg ml^{-1} ketamine + KCl, or 10 μM nifedipine +

KCl. Summarized data are in d (n=5 BSM cell lysates/each time point) and e (n=5 BSM cell lysates/each time point). Data are shown as box and whiskers, center line is median value, box represents 75% of the data, and whiskers indicate minimum and maximum values. Data are analysed by two tailed Student's t test. P < 0.05 is considered to be significant and P values are given above the bars. Source data are provided as a Source Data file.

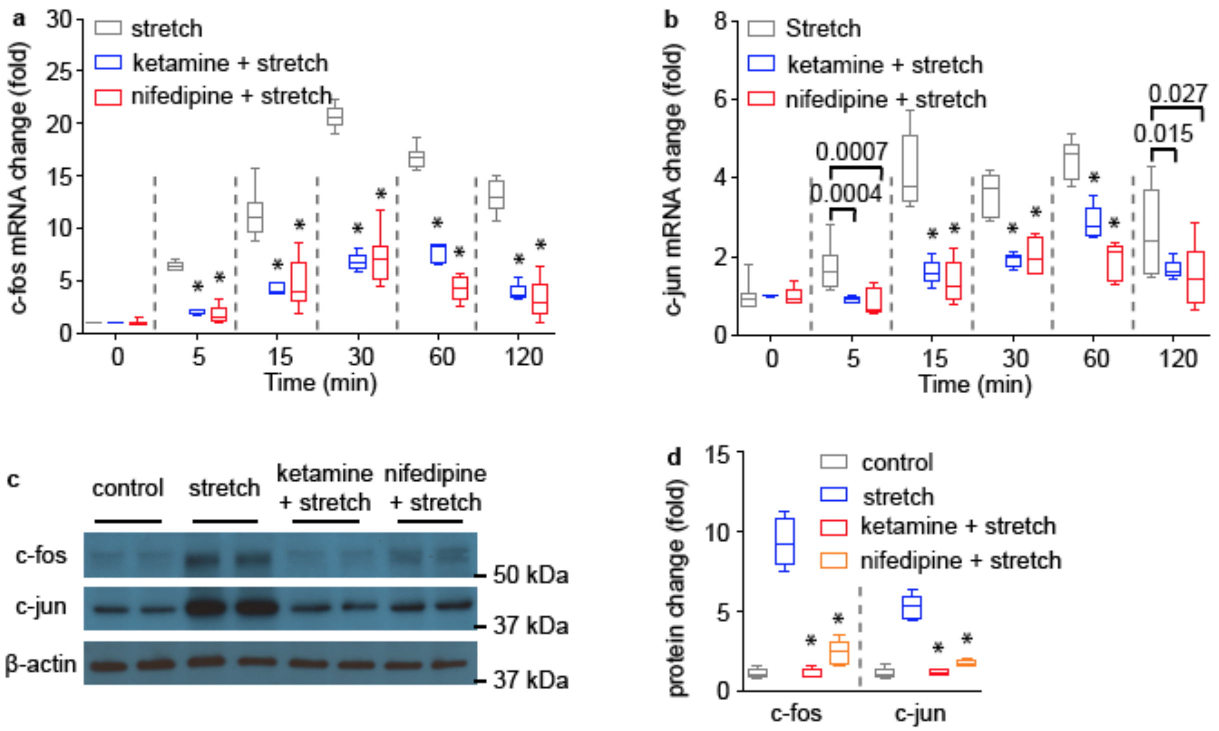

Supplementary figure 10. Ketamine inhibits stretch-induced upregulation of c-fos and c-jun. 2 mm wide mouse BSM strips cut longitudinally and stretched to ~1.5 cm length were pinned on a SYLGARD 184 silicon pad in physiological saline solution for 60 mins. Unstretched bladders served as controls. Bladder tissues were then lysed for preparation of mRNA and protein. a and b (n=9 BSM strip /each time point for all groups) depict upregulation of c-fos and c-jun mRNA levels upon mechanical

stretch, both inhibited by 100 μg ml^{-1} ketamine and by 10 μM nifedipine. c shows representative c-fos/c-jun immunoblots from the experiments summarized in d (n=5 BSM strip lysates for each groups). Data are shown as box and whiskers, center line is median value, box represents 75% of the data, and whiskers indicate minimum and maximum values. Data are analysed by two tailed Student's t test. $P < 0.05$ is considered to be significant and P values are given above the bars. * indicates $P < 0.0001$. Source data are provided as a Source Data file.

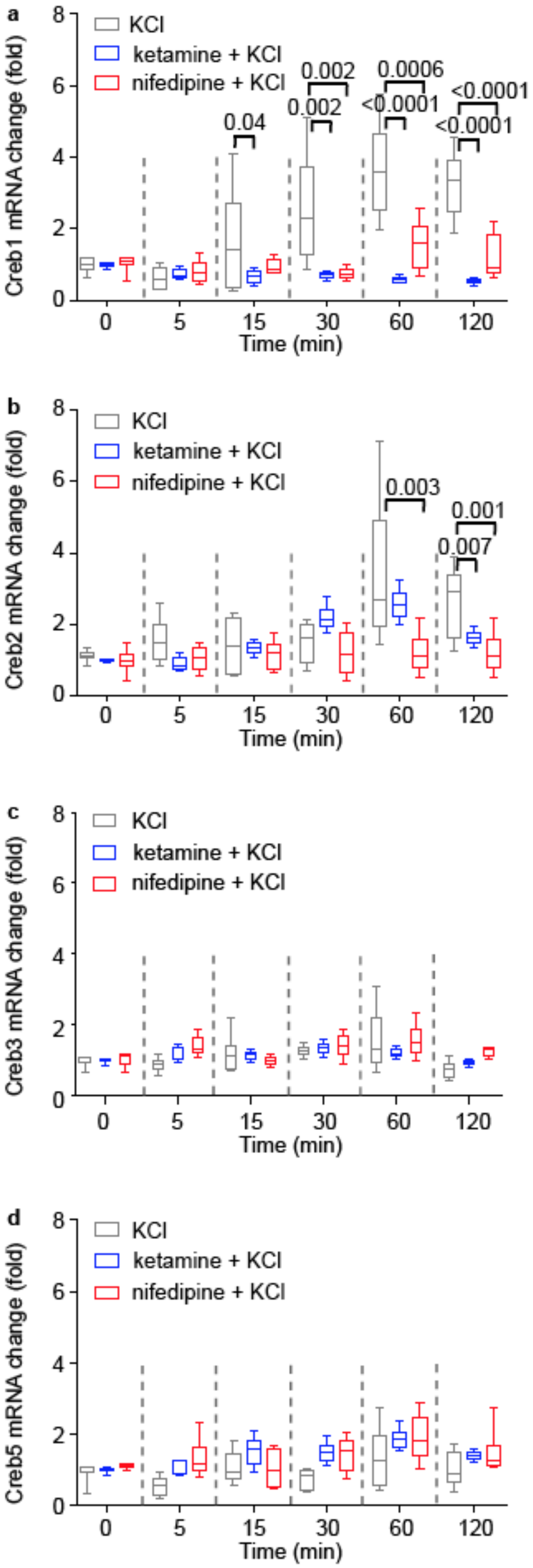
a
Creb1 mRNA change (fold)
KCl
ketamine + KCl
nifedipine + KCl
0.04
0.002
0.002
0.0006
<0.0001
<0.0001
<0.0001
Time (min)
b
Creb2 mRNA change (fold)
KCl
ketamine + KCl
nifedipine + KCl
0.003
0.001
0.007
Time (min)
c
Creb3 mRNA change (fold)
KCl
ketamine + KCl
nifedipine + KCl
Time (min)
d
Creb5 mRNA change (fold)
KCl
ketamine + KCl
nifedipine + KCl
Time (min)

Supplementary figure 11. Ketamine inhibits Cav1.2-stimulated increases in *Creb* family mRNA levels in mouse BSM cells. Mouse BSM strips were subjected to Cav1.2 activation by 50 mM KCl treatment for 0 – 120 min and then lysed for mRNA preparation. Changes in *Creb* family (a-d, n=9 BSM strips at each time point) mRNA levels were measured by quantitative RT-PCR. The increase in creb1 mRNA following treatment with 50 mM KCl was inhibited by 100 µg/ml ketamine and by 10 µM nifedipine treatment. Data are shown as box and whiskers, where center line is median value, box represents 75% of the data, and whiskers indicate minimum and maximum values. Data are analysed by two tailed Student's t test. $P < 0.05$ is considered to be significant and P values are given above the bars. Source data are provided as a Source Data file.

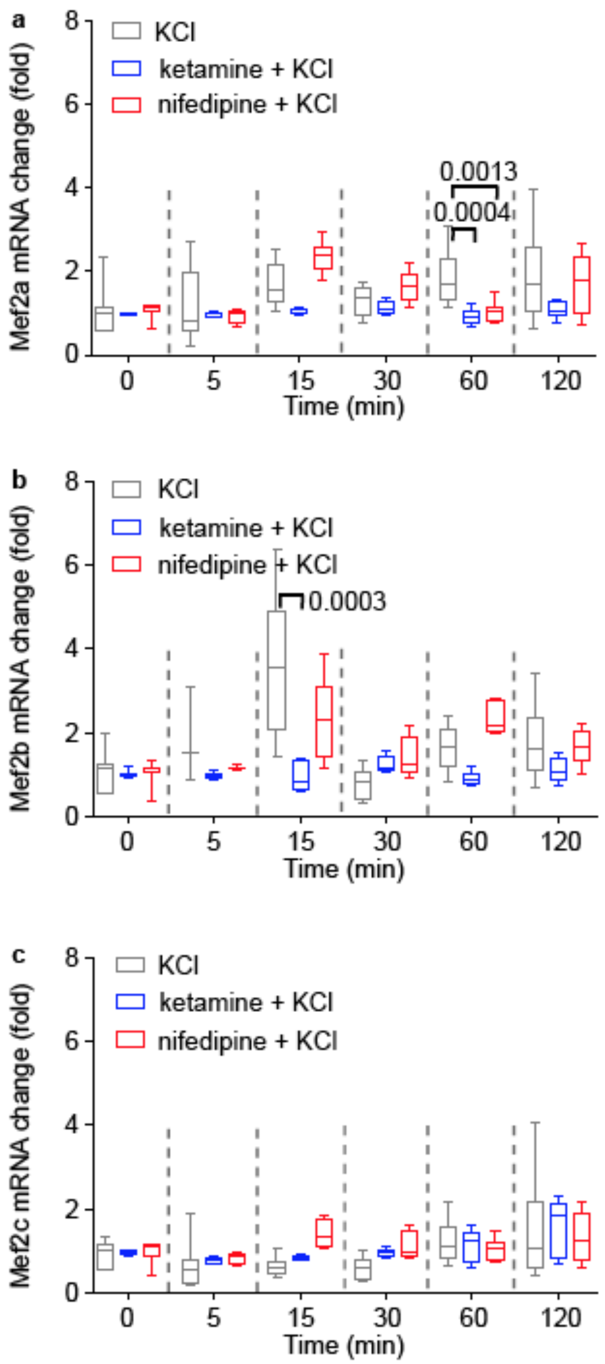

Supplementary figure 12. Ketamine inhibits Cav1.2-stimulated increases in *Mef2* family mRNA levels in mouse BSM cells. Mouse BSM strips were subjected to 50 mM KCl activation of Cav1.2 for 0 – 120 min and then lysed for mRNA. Changes of *Mef* family (a-c, n=9 BSM strips at each time point) mRNA levels were measured by

quantitative RT-PCR. Increases of *Mef2a* and *Mef2b* mRNAs in response to Cav1.2 activation by 50 mM KCl were inhibited by treatment with 100 μg ml^{-1} ketamine or 10 μM nifedipine. Data are shown as box and whiskers, where center line is median value, box represents 75% of the data, and whiskers indicate minimum and maximum values. Data are analysed by two-tailed Student's t test. $P < 0.05$ is considered to be significant and P values are given above the bars. Source data are provided as a Source Data file.

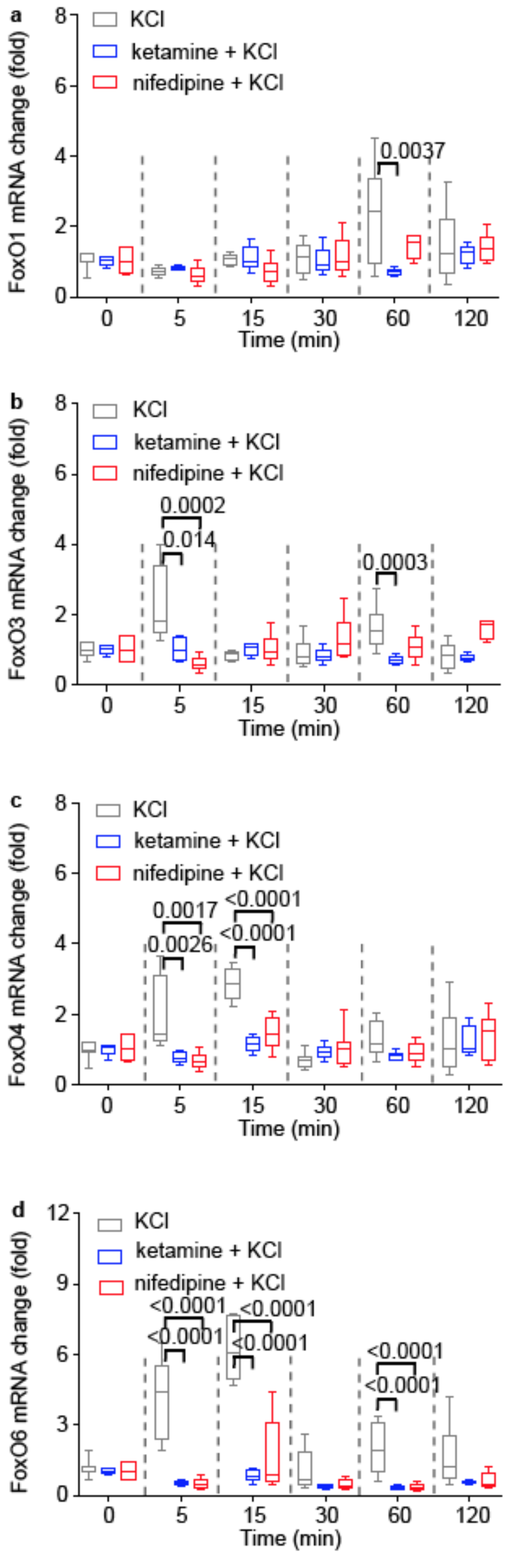

Supplementary figure 13. Ketamine inhibits Cav1.2-stimulated increases in *FoxO* family mRNA levels in mouse BSM cells. Mouse BSM strips were subjected

to 50 mM KCl activation of Cav1.2 for 0 – 120 min and then lysed for mRNA. Changes of *FoxO* family (a-d, n=9 BSM strips at each time point) mRNA levels were measured by quantitative RT-PCR. Increased RNA levels of multiple *FoxO* members in response to 50 mM KCl treatment were inhibited by treatment with 100 μg ml^{-1} ketamine or 10 μM nifedipine. Data are shown as box and whiskers, where center line is median value, box represents 75% of the data, and whiskers indicate minimum and maximum values. Data are analysed by two tailed Student's t test. $P < 0.05$ is considered to be significant and P values are given above the bars. Source data are provided as a Source Data file.

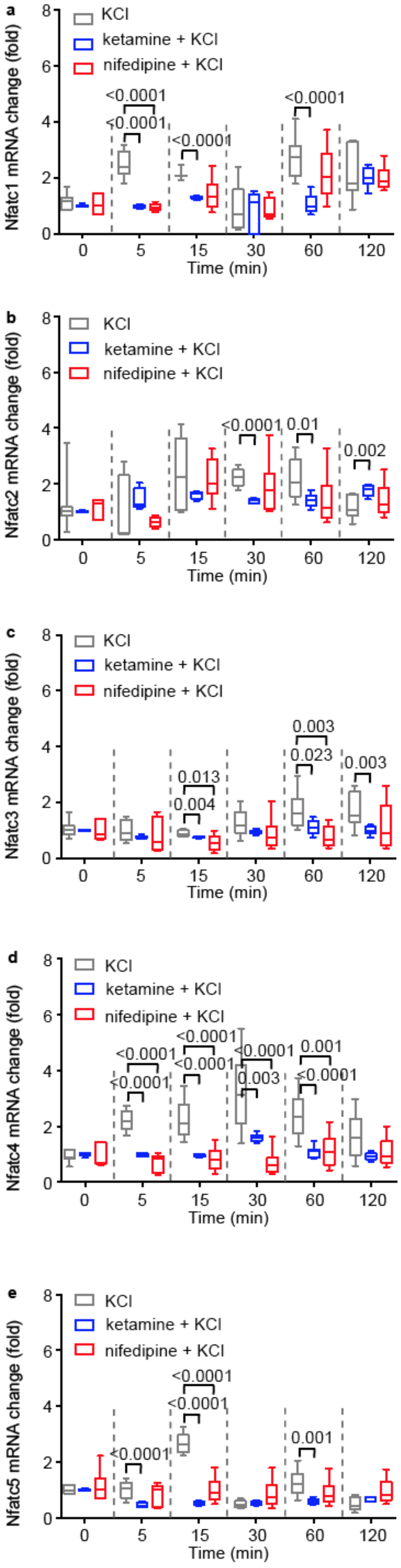
a
b
c
d
e
KCl
ketamine + KCl
nifedipine + KCl
Nfatc1 mRNA change (fold)
Nfatc2 mRNA change (fold)
Nfatc3 mRNA change (fold)
Nfatc4 mRNA change (fold)
Nfatc5 mRNA change (fold)
Time (min)
0
5
15
30
60
120
0
2
4
6
8
<0.0001
0.01
0.002
0.013
0.004
0.003
0.023
0.001

Supplementary figure 14. Ketamine inhibits Cav1.2-stimulated increases in *NFAT* family mRNA levels in mouse BSM cells. Mouse BSM strips were subjected to 50 mM KCl activation of Cav1.2 for 0 – 120 min and then lysed for mRNA. Changes of *Nfat* family (a-e) mRNA levels were then measured by quantitative RT-PCR (n=9 BSM strips at each time point). *Nfatc4* mRNA levels increased by 50 mM KCl were inhibited by treatment with ketamine (100 μg ml^{-1}) or nifedipine (10 μM). Data are shown as box and whiskers, where center line is median value, box represents 75% of the data, and whiskers indicate minimum and maximum values. Data are analysed by two tailed Student's t test. P < 0.05 is considered to be significant and P values are given above the bars. Source data are provided as a Source Data file.

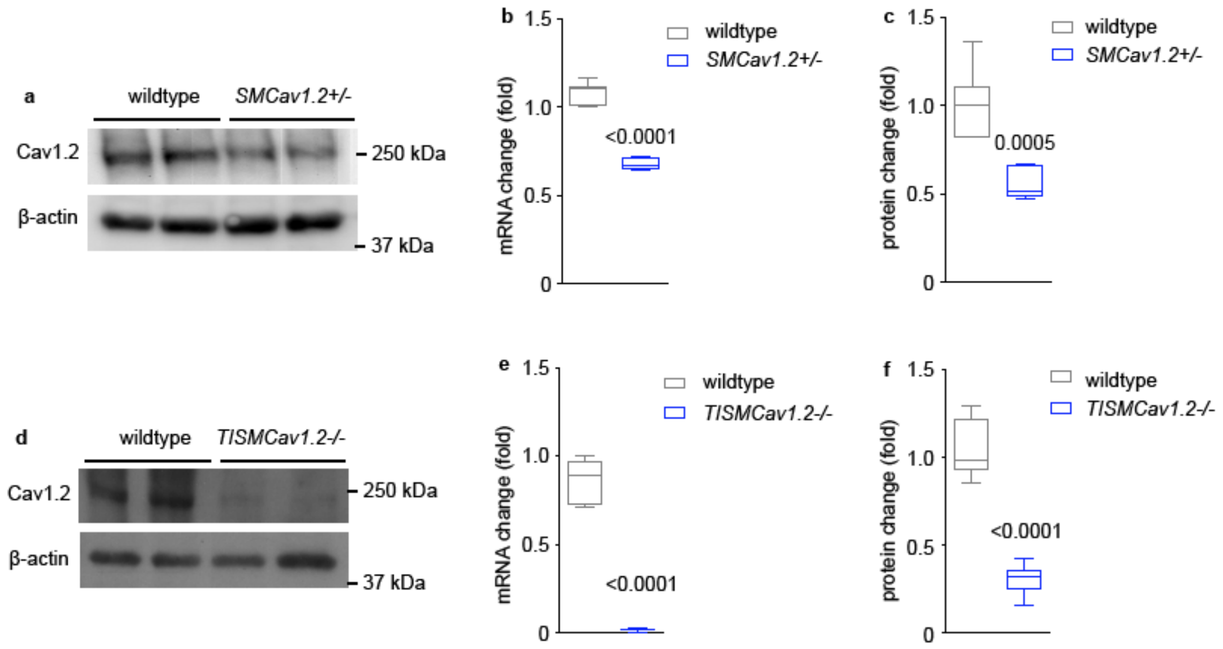

Supplementary figure 15. *SMCav1.2$^{+/-}$* and *TISMCav1.2$^{-/-}$* bladders have reduced levels of Cav1.2 mRNA and protein. Reduced levels of Cav.1.2 protein (c, n=6 mice

for both groups; and f, n=6 mice for both groups) and mRNA (b, n=6 mice for both groups; and e, n=9 mice for both groups) in bladders of *SMCav1.2*$^{+/-}$ and *TISMCav1.2*$^{-/-}$ mice compared to wild type mice. Data are shown as box and whiskers, where center line is median value, box represents 75% of the data, and whiskers indicate minimum and maximum values. Data are analysed by two tailed Student's t test. P < 0.05 is considered to be significant and P values are given above the bars. Source data are provided as a Source Data file.

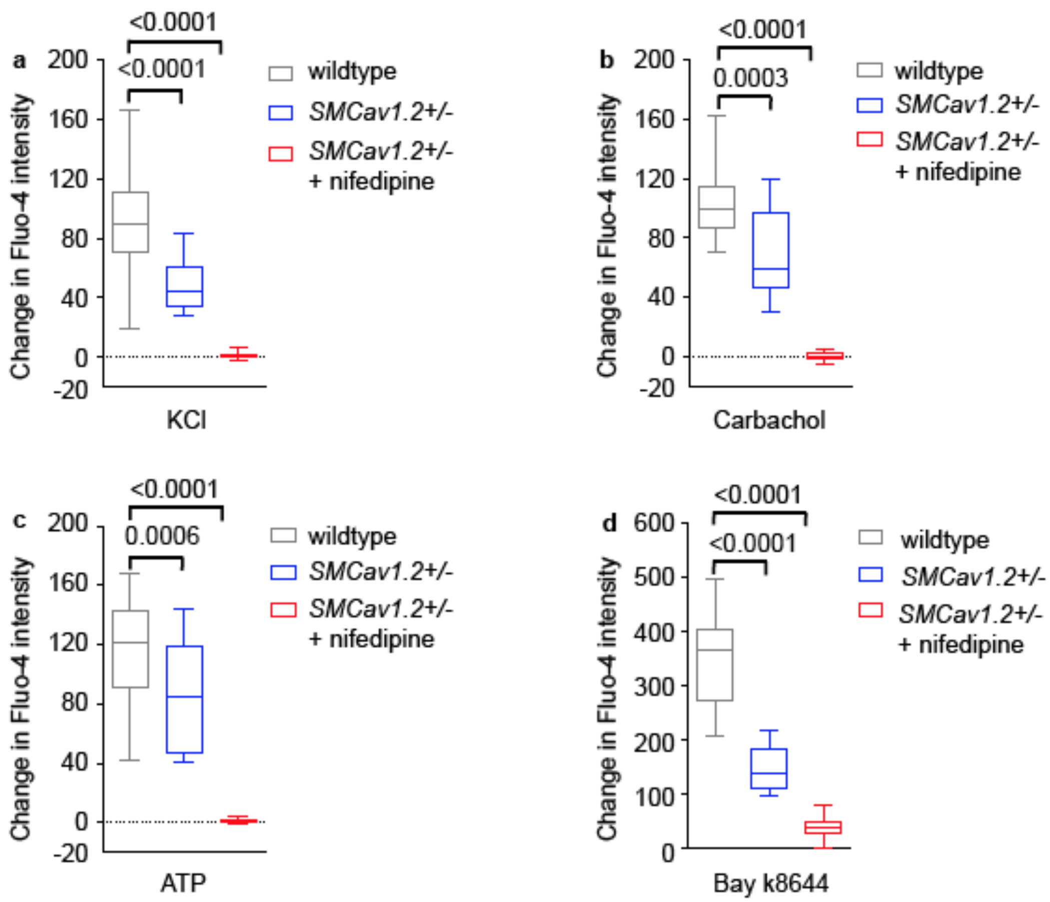

Supplementary figure 16. *SMCav1.2*$^{+/-}$ primary cultured BSM cells exhibit reduced Ca^{2+} influx. a, Intracellular [Ca^{2+}] in 100 mM KCl-stimulated BSM cells from wild type (n=39 BSM cells) and *SMCav1.2*$^{+/-}$ mice in the absence (n=24 BSM cells) and presence

of 10 μM nifedipine (n=36 BSM cells); b, Intracellular [Ca^{2+}] in 100 μM carbachol-stimulated BSM cells from wild type (n=27 BSM cells) and *SMCav1.2*$^{+/-}$ mice in the absence (n=21 BSM cells) and presence of 10 μM nifedipine (n=16 BSM cells); c, Intracellular [Ca^{2+}] in 100 μM ATP-stimulated BSM cells from wild type (n=43 BSM cells) and *SMCav1.2+/-* mice in the absence (n=22 BSM cells) and presence of 10 μM nifedipine (n=39 BSM cells); and d) Intracellular [Ca^{2+}] in 10 nM Bay k8644-stimulated BSM cells from wild type (n=27 BSM cells) and *SMCav1.2*$^{+/-}$ mice in the absence (n=24 BSM cells) and presence of 10 μM nifedipine (n=48 BSM cells). Each condition of stimulated Ca^{2+} influx in *SMCav1.2*$^{+/-}$ mice was inhibited by 10 μM nifedipine. Data are shown as box and whiskers, where center line is median value, box represents 75% of the data, and whiskers indicate minimum and maximum values. Data are analysed by two tailed Student's t test. $P < 0.05$ is considered to be significant and P values are given above the error bars. Source data are provided as a Source Data file.

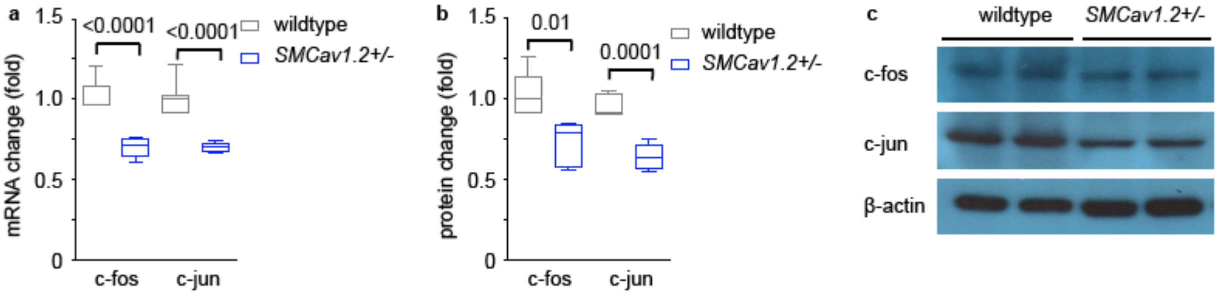

Supplemental figure 17. *SMCav1.2*$^{+/-}$ mouse bladders show decreased levels of *c-fos* and *c-jun* mRNAs and proteins. a, wild type and *SMCav1.2*$^{+/-}$ mouse bladders were lysed for mRNA preparation and subjected to quantitative RT-PCR to determine mRNA levels of *c-fos* and *c-jun* (n=9 mice bladder mRNA extracts for both genotypes).

b, quantitation of c-fos and c-jun immunoblot analysis (n=5 bladder lysates for both genotypes. c, representative immunoblot from two independent experiments. Data are shown as box and whiskers, where center line is median value, box represents 75% of the data, and whiskers indicate minimum and maximum values. Data are analysed by two tailed Student's t test. $P < 0.05$ is considered to be significant and P values are given above the error bars. Source data are provided as a Source Data file.

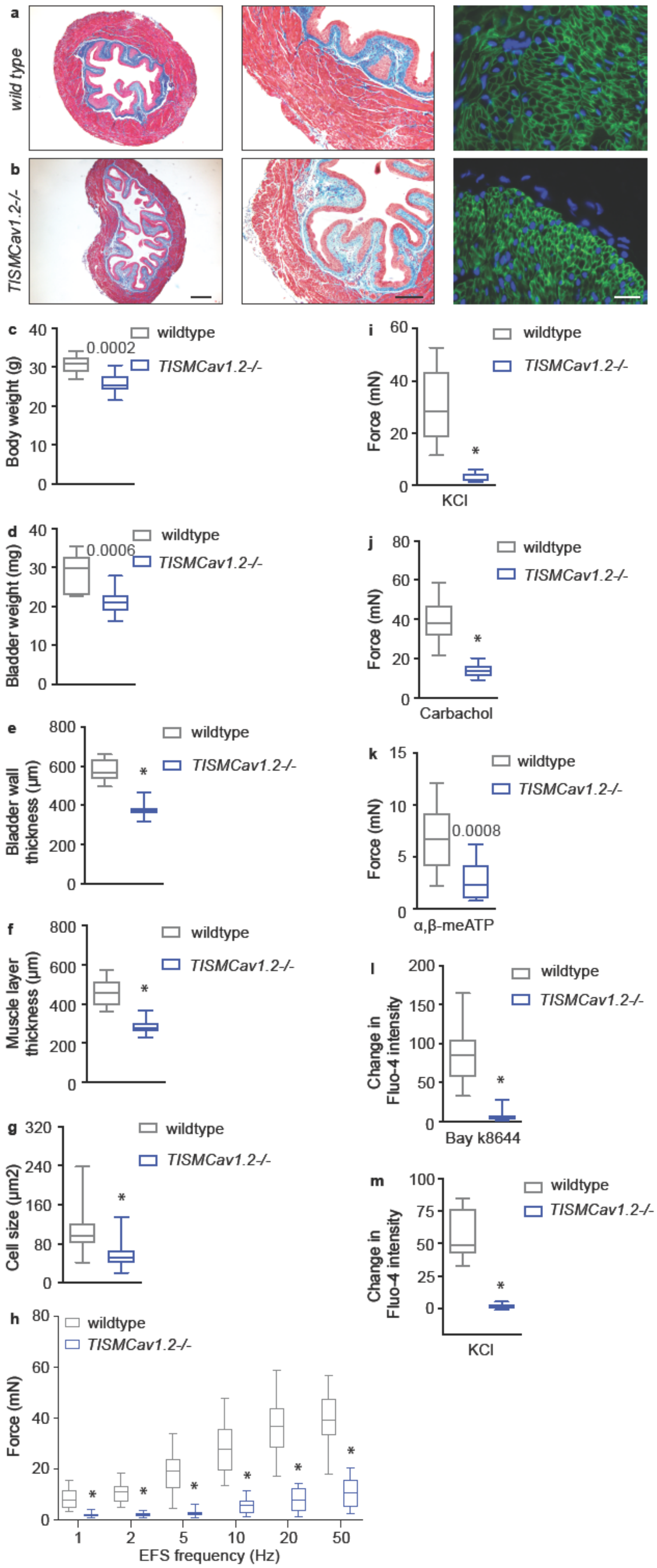
a
wild type
b
TISMCav1.2-/-
c
Body weight (g)
0.0002
wildtype
TISMCav1.2-/-
d
Bladder weight (mg)
0.0006
e
Bladder wall thickness (µm)
f
Muscle layer thickness (µm)
g
Cell size (µm2)
h
Force (mN)
EFS frequency (Hz)
i
KCl
j
Carbachol
k
0.0008
α,β-meATP
l
Change in Fluo-4 intensity
Bay k8644
m
KCl

Supplementary figure 18. *TISMCav1.2$^{-/-}$* mice exhibit abnormal BSM morphology and contractility. a and b are representative images from three independent experiments: Masson's trichrome stained bladder sections show that *TISMCav1.2$^{-/-}$* mouse bladders are floppy with a thinner BSM layer. Scale bars from left to right are 400 μm, 200 μm, and 20 μm, respectively. Summarized data are shown in c – f. c and d (n=10 mice for both wildtype and *TISMCav1.2$^{-/-}$* groups) show that *TISMCav1.2$^{-/-}$* mice have lower body weight and bladder weight. e (n=10 mice for wildtype group and n=14 mice for *TISMCav1.2$^{-/-}$* group) and f (n=14 mice for both wildtype and *TISMCav1.2$^{-/-}$* groups) show that *TISMCav1.2$^{-/-}$* mice have reduced bladder wall and correspondingly lower muscle layer thickness. Individual BSM cells were subjected to immunofluorescence staining with anti-β1 integrin (green) and DAPI for nuclei (blue). BSM cell cross sectional area was quantitated using Fiji software. *TISMCav1.2$^{-/-}$* mouse BSM cell size was significantly smaller (g, n=113 BSM cells for wildtype group and n=78 BSM cells for *TISMCav1.2$^{-/-}$* group). *TISMCav1.2$^{-/-}$* mice BSM showed reduced contraction force in response to EFS (h, n=19 BSM strips for wildtype and n=18 BSM strips for *TISMCav1.2$^{-/-}$*), to KCl (i, n= 16 BSM strips for wildtype and n=11 BSM strips for *TISMCav1.2$^{-/-}$*), to carbachol (j, n= 19 BSM strips for wildtype and n=11 BSM strips for *TISMCav1.2$^{-/-}$*), and to α,β-meATP (k, n= 16 BSM strips for wildtype and n=11 BSM strips for *TISMCav1.2$^{-/-}$*). Primary cultured *TISMCav1.2$^{-/-}$* BSM cells exhibit diminished elevation of intracellular [Ca^{2+}] in response to stimulation with Bay K8644 (l: n= 21 BSM cells for wildtype and n=18 BSM cells for *TISMCav1.2$^{-/-}$*) or with KCl (m: n=15 BSM cells for wildtype and n=26 BSM cells for *TISMCav1.2$^{-/-}$*). Data are shown as box and whiskers, center line is median value, box represents 75% of the

data, and whiskers indicate minimum and maximum values. Data are analysed by two-tailed Student's t test. P < 0.05 is considered to be significant and P values are given above the error bars. * indicates P < 0.0001. Source data are provided as a Source Data file.

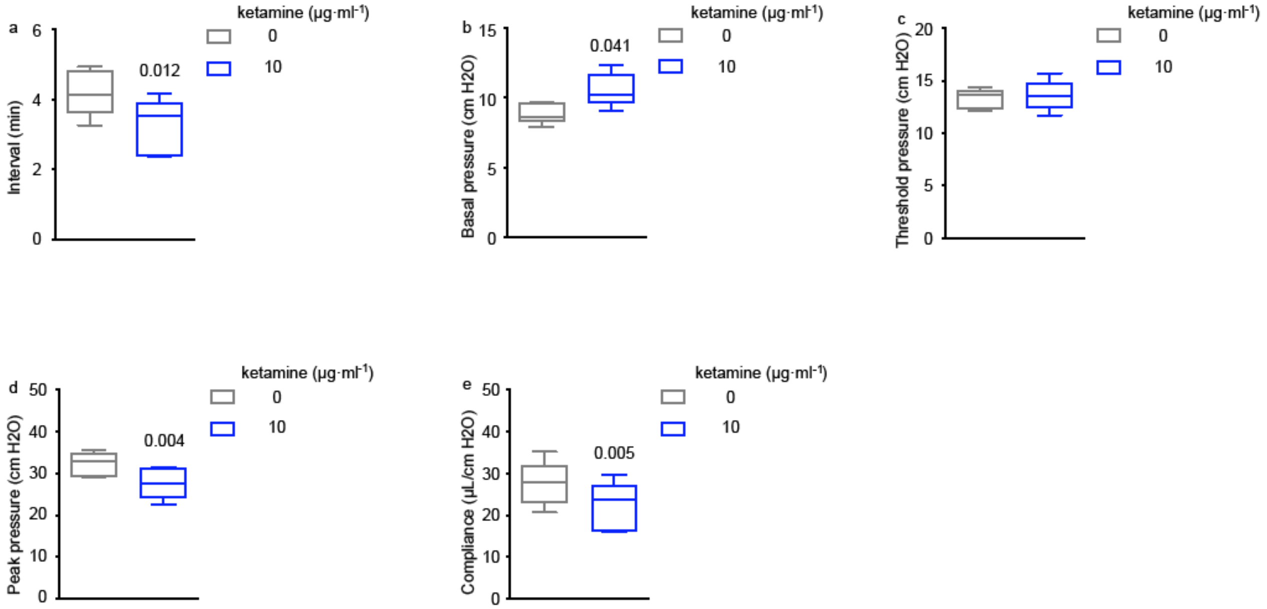

Supplementary figure 19. Ketamine impairs urodynamics in a way resembling ketamine cystitis in humans. Intravesical infusion of ketamine (10 µg ml^{-1}) during CMG decreased voiding interval (a), peak pressure (d), and compliance (e), while increasing basal pressure (b) and leaving threshold pressure unchanged (c). n=5 mice; data are shown as box and whiskers, center line is median value, box represents 75% of the data, and whiskers indicate minimum and maximum values. Data are analysed by paired Student's t test. P < 0.05 is considered to be significant and P values are given above the error bars. Source data are provided as a Source Data file.

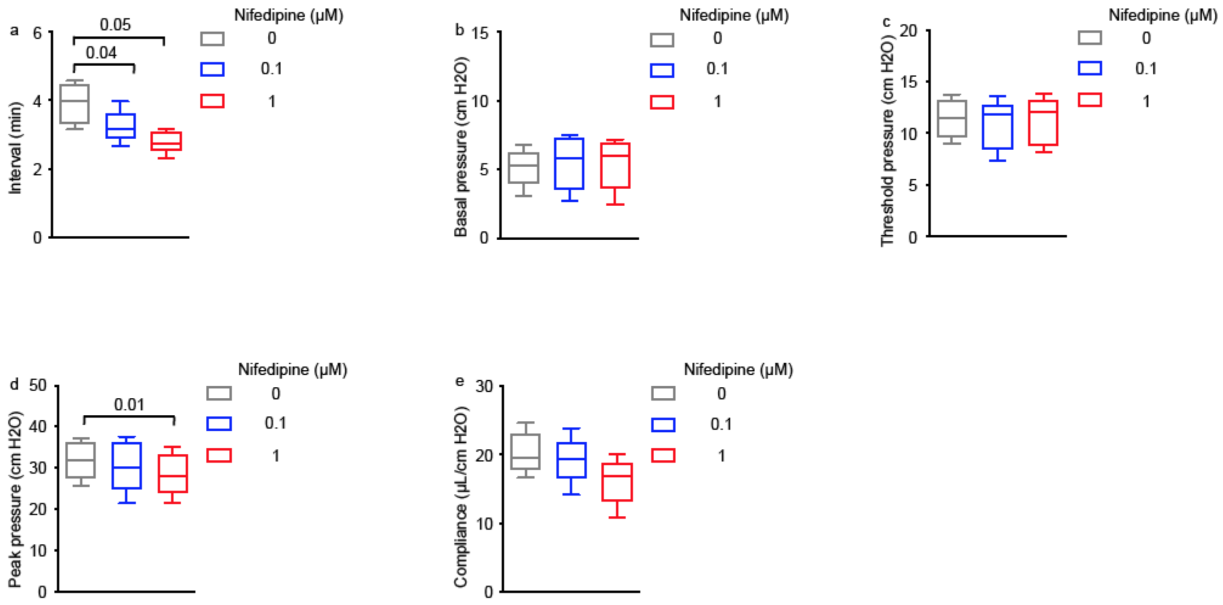

Supplementary figure 20. Nifedipine impairs urodynamics in a way resembling ketamine cystitis in humans. Intravesical infusion of nifedipine (0.1 μM, 1 μM) during CMG decreased voiding interval (a), peak pressure (d), and compliance (e). n=5 mice; data are shown as box and whiskers, center line is median value, box represents 75% of the data, and whiskers indicate minimum and maximum values. Data are analysed by paired Student's t test. $P < 0.05$ is considered to be significant and P values are given above the error bars. Source data are provided as a Source Data file.
