# Supplementary material for: Disruption of Cav1.2-mediated signaling is a pathway for ketamine-induced pathology
Source: Nat Commun. 2020 Aug 28;11:4328. doi: 10.1038/s41467-020-18167-4 (PMC7455701; doi:10.1038/s41467-020-18167-4)
Supplement: Supplementary file 3 — Reporting Summary [file 41467_2020_18167_MOESM3_ESM.pdf]

nature research

Corresponding author(s): Weiqun Yu

Last updated by author(s): 06/16/2020

Reporting Summary

Nature Research wishes to improve the reproducibility of the work that we publish. This form provides structure for consistency and transparency in reporting. For further information on Nature Research policies, see our Editorial Policies and the Editorial Policy Checklist.

Statistics

For all statistical analyses, confirm that the following items are present in the figure legend, table legend, main text, or Methods section.

n/a	Confirmed	
☐	☒	The exact sample size (n) for each experimental group/condition, given as a discrete number and unit of measurement
☐	☒	A statement on whether measurements were taken from distinct samples or whether the same sample was measured repeatedly
☐	☒	The statistical test(s) used AND whether they are one- or two-sided<br>*Only common tests should be described solely by name; describe more complex techniques in the Methods section.*
☐	☒	A description of all covariates tested
☐	☒	A description of any assumptions or corrections, such as tests of normality and adjustment for multiple comparisons
☐	☒	A full description of the statistical parameters including central tendency (e.g. means) or other basic estimates (e.g. regression coefficient) AND variation (e.g. standard deviation) or associated estimates of uncertainty (e.g. confidence intervals)
☐	☒	For null hypothesis testing, the test statistic (e.g. F, t, r) with confidence intervals, effect sizes, degrees of freedom and P value noted<br>*Give P values as exact values whenever suitable.*
☒	☐	For Bayesian analysis, information on the choice of priors and Markov chain Monte Carlo settings
☒	☐	For hierarchical and complex designs, identification of the appropriate level for tests and full reporting of outcomes
☒	☐	Estimates of effect sizes (e.g. Cohen's d, Pearson's r), indicating how they were calculated

Our web collection on statistics for biologists contains articles on many of the points above.

Software and code

Policy information about availability of computer code

Data collection	Myograph and cystometrogram data were collected through LabChart software V8.1.3.<br>Calcium imaging data were collected by MetaFluor Imaging Software (6.1r5, Molecular Devices).<br>Oocyte and muscle cell signals were amplified by two-electrode voltage-clamp using a Geneclamp 500 amplifer. The currents were sampled using a Digidata 1322A board.<br>Quantitative PCR for gene detection was performed and collected with 7300 real-time PCR system (v2.3).
Data analysis	Voiding spot Images were analyzed by UrineQuant (V1.01) software developed by us in collaboration with the Harvard Imaging and Data Core.<br>Myograph and Cytometrogram data were analyzed by LabChart software V8.1.3.<br>MetaFluor Imaging Software (6.1r5) was used to analyze data of the calcium image.<br>Data of the oocytes and muscle cell currents were analyzed using pClamp 8.0 software (Axon Instruments).<br>Gene expression levels were analyzed by 7300 real-time PCR system (V2.3).<br>Western blot bands were quantified by Fiji software (V2.0.0).<br>GraphPad Prism 8<br>Adobe illustrator CS3<br>Adobe Photoshop CS4<br>Microsoft Excel V16.16.22

For manuscripts utilizing custom algorithms or software that are central to the research but not yet described in published literature, software must be made available to editors and reviewers. We strongly encourage code deposition in a community repository (e.g. GitHub). See the Nature Research guidelines for submitting code & software for further information.

Data

Policy information about availability of data

All manuscripts must include a data availability statement. This statement should provide the following information, where applicable:

- Accession codes, unique identifiers, or web links for publicly available datasets
- A list of figures that have associated raw data
- A description of any restrictions on data availability

Nucleotide sequences of mouse Cav1.2 subunits α1 (Catalog no. 26572), rat α2δ1 (Catalog no. 26575), and rat β3 (Catalog no. 26574) are available at www.addgene.org. The datasets generated and/or analysed during the current study are available from the corresponding author on reasonable request. The source data underlying Figs 1b, f, h, 2c-h, 3d–h, 4d, e, h, 5d-h, 6a-d, f, 7d, b, 8c-m, 9e-l, 10c-l and Supplementary Figs2a-e, 3d-h, 4c-f, 5a-e, 6a-f, 7d, g, h, 8a-f, 9a-e, 10a-d, 11a-d, 12a-c, 13a-d, 14a-e, 15a-f, 16a-d, 17a-c, 18c-m, 19a-e, and 20a-e are provided as a Source Data file.

Field-specific reporting

Please select the one below that is the best fit for your research. If you are not sure, read the appropriate sections before making your selection.

[x] Life sciences [ ] Behavioural & social sciences [ ] Ecological, evolutionary & environmental sciences

For a reference copy of the document with all sections, see nature.com/documents/nr-reporting-summary-flat.pdf

Life sciences study design

All studies must disclose on these points even when the disclosure is negative.

Sample size	Sample size was chosen based on previously reports conducted by us and many other labs in the field or determined to be adequate the magnitude and consistency of measurable differences between groups (e.g. Hao et al, JCI Insight 4 (16), (2019); Zhang et al, Nature Communications 7 (2016). Voiding spot assay data are based on a minimum of 10 animals each group (Chen et al, AJP renal 313(6), F1274-1280).
Data exclusions	On principle, data were only excluded for failed experiments, reasons for which included surgical failures in cystometrogram studies, suboptimal muscle contraction force after equilibrium in myography studies in normal control mice . These are pre-established exclusions.
Replication	Experiments were repeated at least two to three times with same conditions, often with multiple sample numbers each time. The exact number of repeats are given in the figure legend.
Randomization	Under matching conditions for age and sex, mice were randomly assigned to control or treatment groups.
Blinding	For most experiments, no blinding was done in the data collection, analysis and quantifications. However, quantification of voiding spot assay was done in an automated fashion using UrineQuant (V1.01) software. Quantification of cystometrogram and myography were done by LabChart software. Most of the data were generated and analysed by automatic softwares as described in data analysis.

Reporting for specific materials, systems and methods

We require information from authors about some types of materials, experimental systems and methods used in many studies. Here, indicate whether each material, system or method listed is relevant to your study. If you are not sure if a list item applies to your research, read the appropriate section before selecting a response.

Materials & experimental systems

n/a	Involved in the study	
[ ]	[x]	Antibodies
[x]	[ ]	Eukaryotic cell lines
[x]	[ ]	Palaeontology and archaeology
[ ]	[x]	Animals and other organisms
[x]	[ ]	Human research participants
[x]	[ ]	Clinical data
[x]	[ ]	Dual use research of concern

Methods

n/a	Involved in the study	
[x]	[ ]	ChIP-seq
[x]	[ ]	Flow cytometry
[x]	[ ]	MRI-based neuroimaging

Antibodies

Antibodies used	α1C antibody (1:1000, AB5156) was purchased from Millipore; C-Fos (1:1000, CST, 2250) and C-Jun (1:1000, CST, 9165) antibodies were purchased from Cell Signaling Technology (Danvers, MA, USA). Ki67 (1:200, ab15580) was purchased from abcam. Beta 1 integrin antibody (1:100, 550531) was purchased from BD. Pharmingen. Rabbit anti-NR1 antibody was from Alomone Lab (#AGC-001). Goat anti rabbit Ig-HRP (1:5000, #1705046) was purchased from Bio-Rad. Donkey anti-rabbit (A32790) and Donkey anti-

	rat (A21208) Alexa 488-conjugated secondary antibodies were purchased from Invitrogen.
Validation	α1C antibody was validated by western blot using lysates from the bladder of α1C gene knock out mice and oocytes expressed α1C protein. Detailed information is shown in the manuscript.<br>Manufacture validated the C-jun antibody by western blot using extracts of NIH/3T3 and SK-N-MC cells, we further validated it by western blot using lysates from the mice bladder, the band we get is at the expected molecular weight. Detailed information can be accessed in https://www.cellsignal.com/products/primary-antibodies/c-jun-60a8-rabbit-mab/9165?site-search-type=Products and our manuscript.<br>Manufacture validated the C-fos antibody by western blot using extracts of HeLa and H-4-II-E cells, we further validated it by western blot using lysates from the mice bladder, the band we get is at the expected molecular weight. Detailed information can be accessed in https://www.cellsignal.com/products/primary-antibodies/c-fos-9f6-rabbit-mab/2250?site-search-type=Products and our manuscript.<br>Manufacture validated Ki67 antibody by ICC/IHC and IHC-P, were further tested by IF. Detailed information can be accessed in https://www.abcam.com/ki67-antibody-ab15580.html and our manuscript.<br>Beta 1 integrin antibody was validated by knockout mice tissue in our previous publication: https://www.fasebj.org/doi/full/10.1096/fj.12-223404.<br>Manufacture validated NR1 antibody by western blot, were further validated by published article (Alosisi, Nature Communications 8 (2017))

Animals and other organisms

Policy information about studies involving animals; ARRIVE guidelines recommended for reporting animal research

Laboratory animals	All the male and female mice used in this study were in C57BL/6J background and purchased from Charles Rivers or Jackson Laboratory. All the mice used in the study are from 12 -16 weeks of age.<br>SM22α-CreKi (stock 006878, Jackson laboratory)<br>SMMHC-CreERT2 (stock 019079, Jackson laboratory)<br>NR1 floxed mice (stock 005246, Jackson laboratory)<br>α1 subunit floxed mice (stock 024714, Jackson laboratory)<br>Mature female Xenopus were purchased from the Department of Systems Biology, Harvard Medical School.<br>Mice were housed in ventilated cages under a 12:12-h dark–light cycle (light from 07:00 to 19:00) at 22 ± 1°C temperature and 60% humidity with ad libitum access to food and water.
Wild animals	This study did not involve wild animals.
Field-collected samples	This study did not involve field-collected samples
Ethics oversight	All animal studies were performed with the approval of the Beth Israel Deaconess Medical Center Institutional Animal and Use Committee and in adherence to U.S. National Institutes of Health guidelines for animal care and use.

Note that full information on the approval of the study protocol must also be provided in the manuscript.
